# Review on therapeutic targets for COVID-19: insights from cytokine storm

**DOI:** 10.1136/postgradmedj-2020-138791

**Published:** 2020-10-02

**Authors:** Mário Luciano de Mélo Silva Júnior, Lívia Maria Alves de Souza, Renata Ellen Maria Carvalho Dutra, Ramon Gonçalves de Melo Valente, Thayanara Silva Melo

**Affiliations:** Medical School, Centro Universitário Maurício De Nassau, Recife, Brazil; Neurology Unit, Hospital da Restauração, Recife, Brazil; Medical School, Centro Universitário Maurício De Nassau, Recife, Brazil; Medical School, Centro Universitário Maurício De Nassau, Recife, Brazil; Medical School, Centro Universitário Maurício De Nassau, Recife, Brazil; Dentistry post-graduation program, Universidade Federal De Pernambuco, Recife, Brazil

**Keywords:** Virology, Tropical medicine, Pharmacology, General medicine (see Internal Medicine), Basic sciences, Immunology

## Abstract

**Introduction:**

Severe acute respiratory syndrome coronavirus 2 (SARS-CoV-2) has been caused the greatest pandemic of our century. Many of the deaths related to it are due to a systemic inflammatory response, which has been called ‘cytokine storm’.

**Objectives:**

We developed a comprehensive review of the pathophysiology mechanisms of COVID-19 and of the rationale for drugs and therapeutics that have been tested in clinical trials.

**Methods:**

A narrative review of the literature was conducted using PubMed, SciELO, Bireme, Google Scholar and ClinicalTrials.

**Results:**

SARS-CoV-2 has evolutive mechanisms that made it spread all around the globe, as a higher latency period and a lesser lethality than other coronaviruses. SARS-CoV-2 causes a delay in the innate immune response and it disarranges the immune system leading to an overwhelming inflammatory reaction (the ‘cytokine storm’). In this scenario, high levels of interleukins (IL), notably IL-6 and IL-1, create a positive feedback of chemokines and immune responses, and powers pulmonary and systemic tissue damage, leading to capillary leakage and SARS, the main cause of death in patients with COVID-19. On 17 July 2020, there were 1450 entries on ClinicalTrials.gov of ongoing studies on COVID-19. The mechanisms of the main therapeutic approaches were comprehensively reviewed throughout the text. Therapies focus on blocking viral entry (remdesivir, umifenovir, among others) and blocking of immune system for cytokine storm control (IL-1 and IL-6 inhibitors, glucocorticoids, convalescent plasma, among others).

**Conclusions:**

Understanding of action mechanisms of SARS-CoV-2 enables us to direct efforts on effective therapeutic targets. This comprehensive review helps to interpret the clinical results of the several trials ongoing.

## INTRODUCTION

Severe acute respiratory syndrome coronavirus 2 (SARS-CoV-2) is a member of the beta-coronaviridae family, which contains Middle-East respiratory syndrome (MERS)-CoV and SARS-CoV. In common, these viruses are crown-shaped on electron microscopy, cause acute respiratory syndrome in humans and have pandemic potential. In contrast, SARS-CoV-2 has higher incubation periods and reproduction number (R0), and then, despite being less virulent,^1^ it has spread quickly and caused the largest pandemic in the 21st century, with striking impacts on social, economic and health systems.

Although more than 14 million cases of coronavirus disease (COVID-19) have been officially reported around the world, leading to more than 600 thousand deaths (update on 17 July), no specific treatment or vaccine is yet available. In the need for urgent responses to this global threat, 1416 trials addressing COVID-19 therapies are registered as ongoing in ClinicalTrials (update on 17 July).

Severe forms of COVID-19 are related to cytokine storm—an immune-mediated sepsis-like syndrome in which host response to SARS-CoV-2 leads to an overwhelming cytokine release. This is associated with severe hypoxemia, multiple organ failure and coagulation disturbances.^2^ This review aims to shed light on pathophysiological mechanisms of SARS-CoV-2 and to explore the rationale behind the therapeutic targets of clinical trials ongoing.

## METHODS

A narrative review of the literature using ‘cytokine storm’ and ‘COVID-19 or coronavirus’ terms was conducted in PubMed, BVS, SciELO and Google Scholar databases. Articles found were assessed as potential reference sources. Also, we searched in ClinicalTrials for ‘COVID-19’ ongoing studies enrolling potential therapeutic drugs related to pathophysiological mechanisms and cytokine storm. From this, we also researched the same databases for the rationale of using those drugs in COVID-19. Searches were performed up to 17 July 2020.

## VIRAL STRUCTURE AND EPIDEMIOLOGY

SARS-CoV-2 crossed the animal–human barrier coming from bats in China. It is a single-strand positive-sense RNA virus that is composed of four main structural proteins: spikes (S), small envelope, membrane glycoproteins and nucleocapsid protein.^3^ Protein S facilitates the binding of the virus to the ACE2 receptor of host cells. In addition, protein S is broken down by the furin-like protease into two subunits called S1 and S2. S1 is responsible for cell tropism, while S2 acts mediating the fusion of the virus in host cells.^3^

The transmission of SARS-CoV-2 occurs person-to-person, but also through the air, droplets and aerosols, as it reaches the respiratory epithelium. Vertical transmission of SARS-CoV-2 is not established.^4^ Transmission rates are high (R0=2.5) and the viral load may be related to the host’s immune response and disease severity.^5^ In this sense, the idea of social isolation (in parallel to the closure of schools and commerce, and its consequences) may lead to a ‘flattened contamination curve’, which is the current main strategy for containing the spreading of the disease.

Technological and social advances that allow intercontinental travel and trade also promote the spread of diseases across the globe, on a scale never seen before. Over the years, international health emergencies declared by WHO has become more frequent, which is a warning sign for the surveillance systems and control of emergent new diseases^6^ The SARS-CoV-2 pandemic started in December 2019 and it has already reached millions of people and remains a challenge for the world.

## VIRAL MECHANISMS OF ACTION AND CITOKINE STORM

The interaction of SARS-CoV-2 with the ACE2 receptor occurs mainly in the alveolar epithelial type 2 cells, where there is a high concentration of it.^3^ After adhesion, the virus fuses and enters into the cell using proteases such as cathepsin, transmembrane serine protease 2 and AP2-associated protein kinase (AAK1), which is a member of the clathrin-mediated endocytosis regulator family.^1^ Viral multiplication occurs using the genetic machinery of the host and involves the 3-chymotrypsin-like protease (3CL). Then, the infected cells begin to expose pathogen-associated molecular patterns, including viral RNA, that are recognised by the innate immune system, such as dendritic and natural killer cells, and macrophages, through Toll-like receptors and the pathway of nuclear factor kappa light chain enhancer of activated B cells .^7^ This process involves recruiting other cells to contain the infection through cytokines and chemokines in an attempt to eliminate the virus.

‘Cytokine storm’ has been used widely both in the lay media and in technical materials. It refers to dysregulation of the immune system, with a substantial release of pro-inflammatory cytokines, which leads to overwhelming tissue damage.^7, 8, 9^ This is the biochemical equivalent of systemic inflammatory response syndrome. The observation of this type of exaggerated cascade response has been described in graft versus host syndrome, as well as induced by drugs and microorganisms.^8^

**Figure 1:**
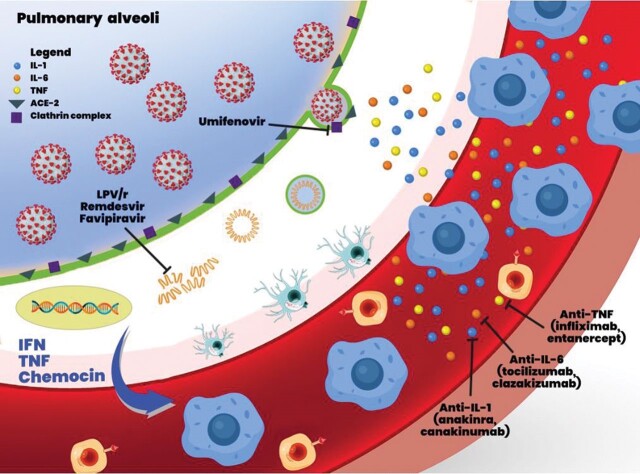
Cytokine storm. Figure shows SARS-CoV-2 contacting, invading and releasing genetic material in an alveolar pulmonary type 2 cell. This may induce cell death, but also leads to exposition of pathogen-associated molecular patterns, which is recognised by leucocyte pattern recognition receptors, both activate innate immunity and cause interferon (IFN), tumour necrosis factor (TNF)-α and chemokines release. This attracts leukocytes that release cytokines (notably IL-1, IL-6 and TNF) and induce tissue damage and cell death, which leads to a vicious cycle of inflammation (cytokine storm) and tissue damage. Some drugs have been studied as therapeutic targets (block to viral entry or to cytokine storm) and are listed in the figure. For further details, see the text. IL, interleukin; LPV/r, lopinavir and ritonavir; SARS-CoV-2, severe acute respiratory syndrome coronavirus 2.

This storm runs with high levels of components of innate immunity against viruses, such as interferons (IFNs), cytokines, chemokines and tumour necrosis factor-α (TNF-α). Cytokines are produced by several cell types in response to activation of pattern recognition receptors (PRRs) or cell death. PRRs identify pathogenic molecular sequences (such as viral RNA) as part of the innate response to viruses. It attracts leukocytes and facilitates diapedesis, which increases the intensity of local inflammation and cell destruction, which in turn produces more cytokines and a chain reaction^8  10^ (figure 1). Chemokines are types of cytokines with potent chemotactic action, and animal studies have observed that lung injury is dependent on their activation and that chemokine levels, such as CXCL10, could be prognostic markers in SARS-CoV-2 infection.^9^

The direct cytopathic effect of SARS-CoV-2 may induce cell death and oedema.^11^ The answer to this type of injury is the release of cytokines like interleukin (IL)-6, IL-8, IL-1β, and granulocyte-macrophage colony-stimulating factor and chemokines like CCL2, CCL5, CXCL10 (previously known as IP-10), CCL-3, along with reactive oxygen species. Such cytokines attract T cells that, in turn, produce TNF-α and IFN, which activate endothelial and dendritic cells that respond by producing more cytokines, especially IL-1 and IL-6.^12^ This positive feedback system is harmful to the host, as it results in the induction of cell apoptosis and necrosis, diffuse alveolar lesion, fibrin deposition, pulmonary fibrosis and SARS syndrome,^7^ which is the main cause of death in patients with COVID-19.^13^

IL-6 has two main receptors, the membrane one (mIL-6R) and the soluble one (sIL-6R). The first is present in leukocytes and when activated they induce a pleiotropic response, via JAK-STAT (Janus kinases and signal transducer and activator of transcription) complex, which includes activation of cellular and innate immunity, as well as Th17 differentiation. When at high levels, IL-6 binds to sIL-6R, which can bind to almost any cell and induce the same mechanism, but now producing vascular endothelial growth factor, CCL-2, IL-8 and more IL-6.^14^ Then, IL-6 is a factor that stands out in most studies as an initial component of the cytokine storm, but also other pro-inflammatory factors such as IL-1, TNF-α and IFN.^12^

Understanding the factors of this amplification can help in the elaboration of strategies to mitigate the tissue injury and, consequently, avoid deaths. What directs a specific individual to trigger a catastrophic inflammatory response or control the infection is still unclear, but probably higher viral loads, delayed or insufficient IFN response (a viral mechanism already presented by SARS-CoV and MERS)^11  15^ and genetic factors play central roles. Recent work by a genomic association pointed out risk variations in several *loci*, among them in the chemokine receptor 6 gene and ABO blood group system, despite not showing differences in human leukocyte antigen genes.^16^

In the context of COVID-19, it was shown that individuals who evolve to severe forms of the disease have, on admission, lower levels of lymphocytes, while higher levels of neutrophils, procalcitonin, C reactive protein and ferritin, as well as TNF-α, IL-2R, IL-6 and IL-8.^17^ Cohorts comparing survivors versus non-survivors also showed that IL-6 and ferritin are significantly increased in the unfavourable evolution group.^18^ IL-10 was also increased in the poor outcome group, which points to a compensatory anti-inflammatory response from the host which may worsen predisposing to secondary infections.^18^

However, the role of immunosuppression in COVID-19 is still not clear, as the effect on virus clearance and the occurrence of secondary infections^19^ can counterbalance the expected bennefits.

## RISK FACTORS AND CLINICAL PRESENTATION

COVID-19 has the potential to cause infection in all humans, with no choice of race, age or social class, but some groups are at higher risk of acquiring a severe presentation. Infected individuals may or not develop the disease. From those who develop symptoms, the vast majority present mild forms; however, about 5% will need intensive medical support, secondary to SARS and multiple organ failure.^20^ The majority, with no or mild symptoms,^21^ are more likely to spread the virus, as they are able to transmit it and tend to take fewer precautions.

The incubation period is 3–19 days.^22^ Initial symptoms of adults with COVID-19 include fever (78%), cough (57%), fatigue (31%), myalgia (17%), sputum (25%), hypo- or anosmis (25%), headache (13%), dizziness (11%), diarrhoea (10%), nausea and vomiting (4%), dysgeusia (4%) and conjunctivitis (2%).^23^ The initial phase can be self-limited, lasting about 7 days, or progress to more severe forms, with oxygen desaturation, respiratory distress and circulatory failure, in a systemic inflammatory response syndrome.

**Figure 2:**
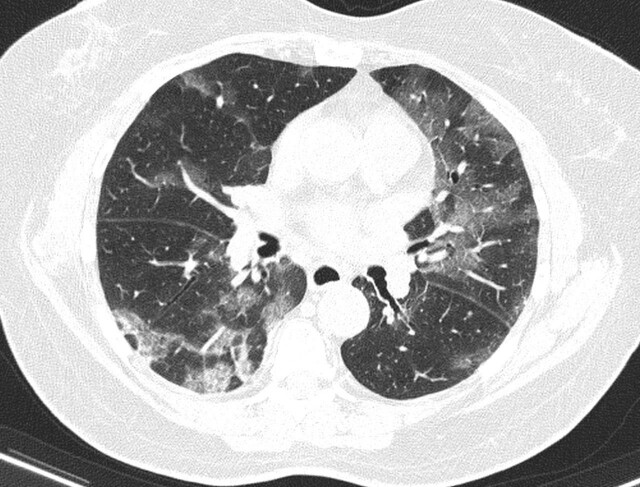
CT of a patient with severe COVID-19. Observation of the bilateral ground glass lesions and interlobular septal thickening, suggestive of COVID-19.

SARS is the main concern of COVID-19, which is secondary to lung injury with dyspnea, hypoxia, hypercapnia, oedema and diffuse alveolar damage requiring invasive ventilatory support.^13^ Alveolar inflammation and increased alveolar-capillary permeability generate image patterns that are nonspecific, such as diffuse bilateral ground glass, but which can increase the diagnostic suspicion (figure 2). Respiratory dysfunction may also be related to diffuse microthrombosis in the pulmonary tree.^24^

There are data suggesting that SARS-CoV-2 can cause myocardial injury and arrhythmias, acute renal failure, coagulopathy, hemodynamic shock,^2^ as well as hepatitis and both central and peripheral nervous system damage (cerebrovascular disease, delirium, seizures, paresthesias).^25^ Central nervous system involvement appears to be related to severe forms of the disease.

Some factors related to the evolution to severe forms of COVID-19 are age over 65 and the presence of comorbidities such as coronary heart disease, diabetes mellitus, hypertension, chronic lung disease, obesity and immunosuppression.^26^ The greater the extent of the lung injury identified in the chest radiographic examination, the greater the chance of admission to the intensive care unit (ICU) and the worse the prognosis^27^; however, the routine performance of CT for diagnostic purposes has not been established. Pregnant women compared to non-pregnant women have a higher risk of developing serious illness due to changes in the woman’s body and in immune system.

## ONGOING TRIALS AND THEIR RATIONALE

### Blocking viral entry

#### Lopinavir and ritonavir

As in HIV, 3-CL protease is important for SARS-CoV-2 viral multiplication, but the inhibition of this protease by lopinavir and ritonavir (LPV/r) occurs at a specific site (C2) for interaction with HIV. However, data indicate that the use of LPV/r in the initial phase of the disease is able to decrease viral shedding duration^28 (not peer-reviewed)^.

A case–control study in which 47 patients were evaluated showed that those on LPV/r reduced their body temperature and had their viral detection negatived faster.^29^ A Chinese study, involving 199 patients observed lower (but not significant) mortality and length of admission in the LPV/r group.^30^

Side effects are nausea, diarrhoea and hepatotoxicity. There are 44 studies with LPV/r registered as ongoing on ClinicalTrials.

#### Remdesivir

Remdesivir inhibits RNA-dependent RNA polymerase (RdRp), leading to an incomplete RNA transcription, with potential against many families of viruses such as Filoviridae, Paramyxoviridae and coronaviruses.

Studies in animals were encouraging, showing respiratory improvement and faster viral clearance.^31^ In humans, a study with 1063 hospitalised individuals showed a reduction of 4 days in recovery time, with no significant difference on mortality reduction (OR =0.70; 95% CI 0.47 to 1.04).^32^ Another study^33^ with 237 patients did not show any positive effect of this drug.

Side effects are nausea, vomiting and elevated liver enzymes.^31^ There are 20 studies in ClinicalTrials on this drug.

#### Favipiravir

It is also a blocker of RdRp, with in vitro benefit over oseltamivir-resistant influenza (the latter has no known activity against SARS-CoV-2). Main adverse effects were high levels of uric acid and liver enzymes, in addition to nausea and vomiting.^30^ There are 15 studies in ClinicalTrials on this drug.

A study involving 80 people with COVID-19 showed a 7-day gain in viral clearance and a faster imaging response in the favipiravir group, but clinical outcomes were not reported.^34^

#### Umifenovir

It impairs the viral fusion with the host cells by inhibition of clathrin-mediated endocytosis. It is used for treatment and prophylaxis of influenza in Russia and China, but it can have an effect on several other viruses.^35^ There are four studies in ClinicalTrials on this drug.

Umifenovir can be better than LPV/r on viral clearance,^36^ while the combination LPV/r plus umifenovir can be higher than LPV/r in terms of clearance viral and radiological improvement.^37^ However, these studies had no control group nor reported clinical outcomes. A study with control group showed no benefit from umifenovir^38^ and the treated group had a longer hospital stay. Adverse effects are mainly gastrointestinal.

### Blocking cytokine storm

#### TNF-α inhibitors

The increase in TNF-α occurs in individuals with severe forms of COVID-19. Studies with SARS-CoV indicate that the interaction of viral S protein with the ACE2 receptor induces the TNF-α-converting enzyme and the local increase in TNF-α both increases tissue damage and facilitates the entry of the virus^39^ in a positive feedback.

Case reports of individuals with exacerbated autoimmune disease (Crohn's and ulcerative colitis) and concurrent COVID-19 treated with infliximab (anti-TNF drug) showed a good clinical and laboratory response.^40  41^ Case series of patients on anti-TNF drugs as etanercept, golimumab and adalimumab^42, 43, 44^ showed a favourable evolution of COVID-19. Patients on anti-TNF-α seemed to evolve less frequently to severe forms of COVID than patients on steroids (15% vs 67%) in an observational study enrolling 198 individuals.^45^

Although these observational data, anti-TNF-α are expensive and yet there are no published trials with these drugs. No case reports with poor outcomes were found in our review, which may be due to a publication bias. ClinicalTrials reports 60 entries for anti-TNF and COVID, mainly with infliximab and adalimumab.

#### Interferon

IFNs are agents produced in response to viral infections through innate immunity, involving the STAT system. A laboratory study in rats with COVID-19 showed that the use of IFN type I inducers resulted in increased viral clearance and decreased tissue inflammation.^46^ Data indicate that the delay in raising the IFN levels in the host runs with a worse prognosis.^11^ There are no case reports on the use of IFNs in COVID-19, but there are 43 records in ClinicalTrials of studies in progress, mainly using IFNs (alpha, beta and lambda) as additional therapy.

IFNs may cause haematological and psychiatric issues.^47^

#### IL-1 inhibitors

IL-1 is produced in response to infectious agents or endogenous signalling of dying cells as part of activated inflammasome. Considering that macrophages and monocytes are important sources of IL-1, that these cells are hyperactivated in COVID-19, that there are high levels of IL-1 in the alveolar lavage of patients with COVID-19,^48^ it was thought that blocking this pathway could limit the cytokine storm. Anti-IL-1 is used to treat other syndromes that develop with cytokine storms, such as Still disease, macrophage activation syndrome and haemophagocytic lymphohistiocytosis.^49^

In a descriptive study of 45 patients with moderate-to-severe COVID, the use of anakinra reduced the number of deaths by 34% in 21 days, but there was no difference regarding mechanical ventilation-free status.^50^ A study with 96 patients with moderate-to-severe disease showed clinical (invasive mechanical ventilation or death) benefit of the group on anakinra in multivariate analysis, without significant side effects.^51^

Although we have not identified publications with the use of other IL-1 inhibitors, there are 15 records in ClinicalTrials of studies with canakinumab and anakinra. Main adverse effects of anti-IL-1 are leucopenia and thrombocytopenia, and elevations of hepatic aminotransferases.

#### IL-6 inhibitors

IL-6 is a central player in the cytokine storm acting as a tissue injury enhancer, through the mechanisms of transcription induction via JAK-STAT and increased pulmonary capillary permeability (sIL-6R), as well as amplifying the immune response (mIL-6R). Data show that higher serum levels of IL-6 are present in more severe cases^52^ and in individuals with a higher viral load.^53^ Increases in IL-6 levels may occur before clinical worsening.^54^

An anti-IL-6 R antibody (tocilizumab) has shown encouraging results in retrospective studies. Twenty-one patients with severe COVID-19 received tocilizumab and showed improvement in fever, respiratory and imaging parameters.^55^ After infusion of tocilizumab IL-6 levels tend to rise and then to fall,^56^ individuals who do not experience a drop in IL-6 levels may have a worse outcome. At least four case reports favourable to the use of tocilizumab are available.^57^ There are also reports of the favourable use of clazakizumab, an anti-IL-6 antibody, which was successful in a case report of a patient with a heart transplant.^58^

Adverse effects are headache, diarrhoea and neutropenia. Its use is still discussed due to the long half-life of tocilizumab (around 20 days), low availability in the clinical setting and the need for testing for tuberculosis before onset,^59^ which limits its use in underdeveloped countries. There are 69 studies ongoing on the ClinicalTrials using sarilumab, tocilizumab (both IL-6R inhibitors), siltuximab and clazakizumab (both IL-6 inhibitors).

#### JAK inhibitors

JAK inhibitors can, in addition to limiting the amplification of the immune response via JAK-STAT, prevent the entry of SARS-CoV-2 into host cells by inhibiting AAK1, which participates in the process of clathrin-mediated endocytosis. Furthermore, there is inhibition of the production of ACE2, which is dependent on JAK,^60^ contributing to the reduction of viral invasion and lung damage secondary to the relative increase in angiotensin.^61^ Baricitinib is the JAK inhibitor with which it is believed to achieve all of these effects in standard doses.^61^

In a retrospective study with 105 patients with COVID-19, 14 were considered severe and treated with ruxolitinib. Of these, 79% reduced the inflammation score, with no adverse effects.^62^ Case series with 12 patients with moderate COVID-19 received baricitinib^63^ and showed clinical and radiological improvement.

The side effects found were reactivation of herpes zoster, thrombosis, diarrhoea, electrolyte imbalance, anaemia and nausea. There are 19 studies at ClinicalTrials involving ruxolitinib and baricitinib.

### Therapies without specific target

#### Corticosteroids

Corticosteroids have a wide and nonspecific anti-inflammatory action. They alter the transcription of mRNA and thus decrease the production of inflammatory mediators. On the other hand, the state of immunosuppression in a fragile respiratory epithelium may delay viral clearance, predispose to secondary infections and clinical deterioration, so the WHO does not recommend the use in patients with severe COVID-19.^64^

A Chinese study of 201 patients (21–83 years old) with severe COVID-19, of which 62 received methylprednisolone (dose not described) and, although they seemed more severe at the start of treatment than controls, had a reduction in mortality (OR=0.38; 95% CI 0.20 to 0.72).^65^ An English study enrolled 2104 patients with COVID-19 to receive dexamethasone 6 mg/day or other therapies. It found a reduction in mortality (OR=0.83; 95% CI 0.75 to 0.93) at 28 days in the subgroup of severe patients (those who needed ventilatory support).^66^ However, studies showed that early use of corticosteroids raised viral load and did not produce any benefit on admission length or duration of symptoms.^67^

Thus, questions such as indication, time of onset and duration of corticosteroids need to be better clarified in well-designed trials. The main side effects are dysglycaemia, necrosis of the femur head and secondary infections. There are 32 studies with corticosteroids (prednisone, methylprednisolone and dexamethasone) registered in ClinicalTrials.

#### Convalescent plasma transfusion

It consists in a transfusion of plasma from recovered patients to individuals who are still infected. The antibodies present in the plasma of the convalescents could promote immunity in the patients.^68^ Convalescent plasma is an approach already used to treat other epidemic outbreaks.^69^ It must contain high effective and specific titers of antibodies that bind to the virus, neutralise it, block its access to uninfected cells and activate effector mechanisms.^68^ The more individuals healed, the more potential donors.

A meta-analysis with 32 studies on severe viral diseases (including MERS and SARS) showed safety and reduced mortality on convalescent plasma recipients.^70^ A pilot study of 10 patients with severe COVID-19 received convalescent plasma in addition to standard treatment. These patients became negative for SARS-CoV-2, improved oxygenation and decreased C-reactive protein; there were no deaths or serious adverse effects.^71^ In a Chinese case series with five patients with severe COVID-19 (all were on invasive ventilation), after 12 days of the transfusion of convalescent plasma, all patients had an undetectable viral load, three were discharged and two remained in stable condition until publication.^72^

Adverse events include transfusion-related lung injury and rash.^70^

#### Nitazoxanide

It is an antiparasitic that has demonstrated in vitro activity against several viruses, including MERS, SARS-CoV and SARS-CoV-2.^73^ The antiviral effect is due to the inhibition of host pathways of viral replication, especially IFN induction, and not of the virus itself. However, there are data on the blocking of influenza virus viral haemagglutinins.^74^

Adverse effects are abdominal discomfort, elevated creatinine and enlarged salivary glands. No case reports were found, but there are eight records in ClinicalTrials about this drug.

#### Ivermectin

Ivermectin is a medication commonly used to treat parasitic infections.^75^ In vitro studies show action against several RNA viruses, such as the dengue virus, zika and yellow fever.^76^ In RNA viruses, ivermectin blocks the nuclear import of viral proteins, limiting viral replication.^77^ Data show that ivermectin reduces 99.8% of SARS-CoV-2 activity in vitro.^78^ There are no case reports on the clinical use of ivermectin on COVID-19.

There are 17 records in ClinicalTrials about COVID-19 and ivermectin.

#### Chloroquine and hydroxychloroquine

A well-established drug in the treatment of malaria and rheumatological diseases which reduces the recruitment of defence cells, because it prevents the recognition of antigens and therefore the production of cytokines.^79^ Hydroxychloroquine (HCQ) is suggested for both prophylaxis and treatment of COVID-19, because in vitro results (blocking viral transport, glycosylation of ACE2 preventing viral binding, alkalinisation of the Golgi complex impairing the formation of virions) were encouraging.^80^ A total of 125 studies with HCQ are listed as ongoing in ClinicalTrials. Several studies with different objectives, methods, populations and outcomes have been published on the use of HCQ in COVID-19. The results are conflicting.^81^

A recent study^82^ with patients in ICU compared HCQ with LPV/r and with control, and observed a mortality rate of around 31% in the three groups. An observational study involving 1376 patients did not identify a benefit in using HCQ over reduced mortality or intubation (OR=1.04, 95% CI 0.81 to 1.32).^83^ A study on prophylaxis after high-risk exposure to SARS-CoV-2 also showed no effect of HCQ.^84^

The main concerns on HCQ use are prolongation of the QT interval, hypoglycaemia and skin rash. Blind and controlled prospective studies have been expected. There are 125 entries in ClinicalTrials.

#### HCQ and azithromycin

There is a synergistic effect between HCQ and azithromycin in vitro against SARS-CoV-2. Azithromycin is also a weak base that accumulates on endosomes in a similar way to HCQ and has immunomodulatory effects. There are 57 studies involving azithromycin listed in ClinicalTrials.

A retrospective study in France evaluated 1061 patients in the early phase of COVID-19 who were given HCQ plus azithromycin. It was observed that 91.7% of the patients had a good clinical outcome^85^; however, there was no control group. Other retrospective study conducted in the USA with 807 patients with COVID-19 who were treated with this combination and observed no benefit in any of the outcomes compared to the control group, but an increase in mortality in the HCQ group with or without azithromycin.^86^

It was observed that the association of these two drugs increases the risk of QT prolongation, tachyarrhythmias and sudden death.^87^ The use of this combination, therefore, must be accompanied by electrocardiographic surveillance and in patients without risk factors for heart disease. Both HCQ and azithromycin are widely accessible and low-cost drugs.

## CONCLUSIONS

Despite the fact that 7 months have passed since the beginning of the SARS-CoV-2 pandemic, no clinical intervention has yet been convincing on improving clinical outcomes in COVID-19. Here we made a comprehensive review of the mechanisms of viral injury and host response, as well as the rationale for conducting trials.

Many prospective randomised studies on COVID-19 interventions are underway, with better designs than the current studies, and in the near future, we should have more results on these different approaches to coping with COVID-19.

**Main messages**

- Death due to COVID-19 is usually a consequence of a cytokine storm.
- The SARS-CoV-2 has immunogenic mechanisms that delay the host response and may induce the cytokine storm.
- Ongoing trials target mainly viral entry mechanisms and immune blocking
- Interleukin-6 has a central role in inflammatory response and tissue damage due to SARS-CoV-2. Larger IL-6 inhibition trials are expected regarding security and efficacy.

**Current research questions**

- Is immune blocking safe in severe COVID-19 setting? Will it be counterbalanced by any secondary infections?
- Are those drugs options for early treatment of non-severe COVID-19 cases?
- Is there superiority in blocking IL-6 or other specific targets?

**Self-assessment questions**

1. What is the main difference between SARS-CoV-2 and the other coronaviruses, specially MERS and SARS-CoV?
  - SARS-CoV-2 is more lethal than MERS
  - SARS-CoV-2 has a lesser reproduction number than SARS-CoV
  - SARS-CoV-2 has a higher incubation period than MERS
2. What are the proteins (of the SARS-CoV-2 and the host, respectively) which binding promotes the viral entry?
  - S and ACE2
  - M and DPP4
  - S and DPP4
  - M and ACE2
3. Considering the effect of the inflammatory amplification cascade, which is the main cytokine in the COVID-19 pathophysiology?
  - TNF
  - IL-1
  - IL-6
  - IFN
4. What is the proposed mechanism of action of umifenovir in the management of COVID-19?
  - Blocking viral multiplication
  - Blocking the viral entry
  - Blocking IL-6
  - Blocking IL-1
5. How baricitinib (a JAK-inhibitor) may improve clinical outcomes in COVID-19?
  - Blocking viral entry only
  - Blocking inflammatory response only
  - Blocking both viral entry and immune response

**Key references**

1. Sakurai A, Sasaki T, Kato S, *et al*. Natural history of asymptomatic SARS-CoV-2 infection. *N Eng J Med* 2020.
2. Rabaan AA, Al-Ahmed SH, Haque S, *et al*. SARS-CoV-2, SARS-CoV, and MERS: a comparative overview. *Le Infez Med* 2020.
3. Wu Z, McGoogan JM. Characteristics of and important lessons from the coronavirus disease 2019 (COVID-19) outbreak in China: summary of a report of 72314 cases from the Chinese Center for Disease Control and Prevention. *JAMA* 2020;323(13):1239–1242.
4. Ellinghaus D, Degenhardt F, Bujanda L, *et al.* Genomewide association study of severe COVID-19 with respiratory failure. *N Engl J Med* 2020;NEJMoa2020283.
5. Ragab D, Salah Eldin H, Taeimah M, Khattab R, Salem R. The COVID-19 cytokine storm; what we know so far. *Front Immunol* 2020;11:1446.

**Answers**

a) False; b) False; c) True
a) True; b) False; c) False; d) False
a) False; b) False; c) True; d) False
a) False; b) True; c) False; d) False
a) False; b) False; c) True
